# A review of the clinicopathologic characteristics of intestinal metaplasia in gastric mucosal biopsies

**DOI:** 10.11604/pamj.2016.23.77.7614

**Published:** 2016-03-10

**Authors:** Olaejirinde Olaniyi Olaofe, Donatus Sabageh, Akinwunmi Oluwole Komolafe

**Affiliations:** 1 Department of Morbid Anatomy and Forensic Medicine, Obafemi Awolowo University Teaching Hospitals Complex, Ile-Ife, Osun State, Nigeria; 2 Department of Morbid Anatomy and Histopathology, Ladoke Akintola University of Technology, Ogbomoso, Oyo State, Nigeria

**Keywords:** Clinicopathologic, characteristics, intestinal metaplasia, gastric biopsies

## Abstract

**Introduction:**

Although it is a well recognized premalignant lesion of the stomach, there is a dearth of information on the clinicopathologic features of gastric intestinal metaplasia in Nigerians. It is, therefore, necessary to study these features and their possible contribution to the development of gastric carcinoma in Nigerians.

**Methods:**

All gastric biopsies with the histo-morphologic features of intestinal metaplasia diagnosed at the department of morbid anatomy and forensic medicine, Obafemi Awolowo university teaching hospitals complex, Ile-Ife, Nigeria between January 2006 and December 2010 were used for the study.

**Results:**

A total of 165 biopsies (21.3% of all gastric biopsies within the study period) with background chronic gastritis and intestinal metaplasia were reviewed. The mean age of patients with intestinal metaplasia was 50.3 years ± 17 standard deviation (SD) while the ages of the patients ranged from 10-100 years. There were 83 males (50.3%) with a mean age of 48.1 ± 18.2 SD years and 95% confidence interval (CI) of 44.1-52.1 years. There were, however, 82 females (49.6%) with a mean age of 52.5 (± 15.8 SD) years and a 95% CI of 49.0-56.0 years. There was no significant association between the histologic type of intestinal metaplasia and the patients’ sex, age groups, severity of chronic gastritis, disease activity or degree of gastric glandular atrophy.

**Conclusion:**

There are no statistically significant differences in the clinicopathologic characteristics of the subtypes of intestinal metaplasia. In majority of patients, progression from intestinal metaplasia to gastric adenocarcinoma probably takes an average of about 7 years.

## Introduction

Intestinal metaplasia of the gastric mucosa is a well recognized premalignant condition of the stomach that arises from a background of well established chronic atrophic gastritis due to various aetiological factors most significant of which is infection of the gastric mucosa by *Helicobacter pylori*. Many studies have since established a strong link between *Helicobacter pylori*, chronic gastritis and intestinal metaplasia. Nevertheless, there are other less frequent causes of this association. These include autoimmune gastritis, alcohol, cigarette smoking, radiation, Chron's disease, uremia and graft-versus-host disease among others [1]. The chronic inflammation is believed to ultimately lead to glandular atrophy and intestinal metaplasia of the gastric mucosa which significantly increases the risk for the development of the intestinal type of gastric carcinoma. This intestinal metaplasia has been classified as complete, incomplete and mixed.

Although there are no clearly defined sequences of genetic alterations that are thought to lead to the development of gastric adenocarcinoma from intestinal metaplasia, mutations of the E-cadherin gene, transforming growth factor receptor ßII, p53, BAX, insulin-like growth factor receptor II have been associated with these events [2, 3]. Efforts are, however, still on going to map out possible pathogenetic pathways where such exist. Various reports have been published on the necessity or otherwise of indicating the histological types of intestinal metaplasia [4–8]. Filipe et *al*, who conducted a cohort study on the subtypes of intestinal metaplasia and their relative risk for the development of gastric carcinoma in a Slovenian population, found some differences in the cancer risk between the various subtypes of intestinal metaplasia [9]. He thus supported the histological sub-typing of intestinal metaplasia as an integral part of the pathological assessment of chronic gastritis in gastric biopsies. [9] El Zimaity *et al* on the other hand, did not support this view since they did not find any significant advantage that doing so might confer on the patient's relative cancer risk [10]. A lot of studies have investigated the possible relationships that exist between gastric carcinoma in Nigerians and some of the well known aetiological factors [11–16]. For example, Sabageh *et al* studied gastric carcinoma in Ile-Ife, Nigeria and found intestinal metaplasia in 56.7% of patients with gastric carcinoma, 40.7% of which were of the intestinal-type. [11] Most of these were found in the gastric antrum. The study emphasized the relationship of gastric carcinoma with intestinal metaplasia of the gastric mucosa. In Nigeria, there is a dearth of information on the pathology of gastric intestinal metaplasia, a well recognized premalignant lesion of the stomach, in contrast to the large volume of work done in other regions of the world which have clearly defined the characteristics of this lesion [11]. It is, therefore, necessary to study the clinical and pathological features of gastric intestinal metaplasia and the role it plays in the development of gastric carcinoma in Nigerians. The main objective of this study was, therefore, to evaluate the age, sex and histo-morphologic characteristics of patients with chronic gastritis and intestinal metaplasia and to compare the mean age of patients with intestinal metaplasia to that of patients with gastric carcinoma as previously described at our centre.

## Methods

This is a five-year retrospective study of all gastric biopsies with histologic features of chronic gastritis and intestinal metaplasia received at the Department of Morbid Anatomy and Forensic Medicine, Obafemi Awolowo University Teaching Hospitals Complex, Ile-Ife, Nigeria between January 1, 2006 to December 31, 2010. The patients’ original request cards were reviewed and the demographic data extracted. The surgical pathology reports were scrutinized while the histology slides were reviewed for details of the gross and microscopic characteristics respectively. The microscopic features assessed included the severity of the chronic inflammation, degree of specialized glandular atrophy, type of intestinal metaplasia and the presence or absence of dysplasia. The severity of chronic inflammation was graded as mild, moderate or marked according to the upgraded Sydney System [16]. The glandular atrophy was graded as absent, mild, moderate or marked using the same system. In addition, intestinal metaplasia was classified as complete or incomplete using the same updated Sydney system. The presence or absence of dysplasia was also assessed and classified as mild, moderate or marked where present. The data was analyzed for differences in proportion, using Chi square (p is significant at <0.05), with SPSS version 15.0. Student t-test of equality of means was also performed. The data was presented as a table and as charts.

## Results

**Figure 1:**
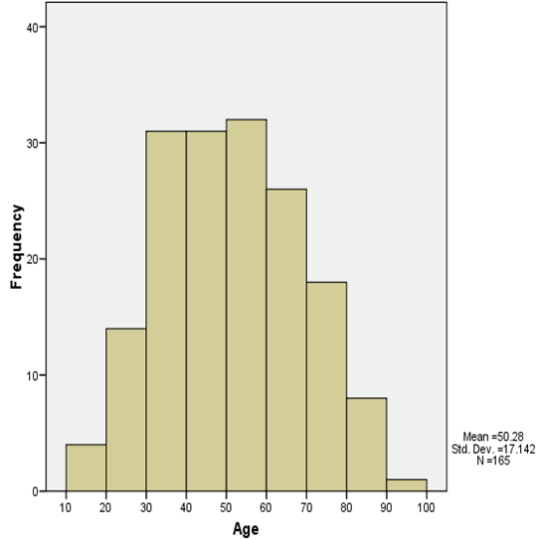
Age distribution of patients

**Figure 2:**
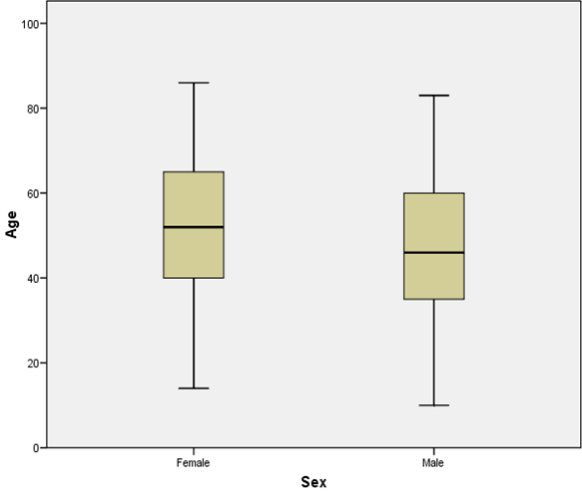
Box plot of patients’ age and sex

**Figure 3:**
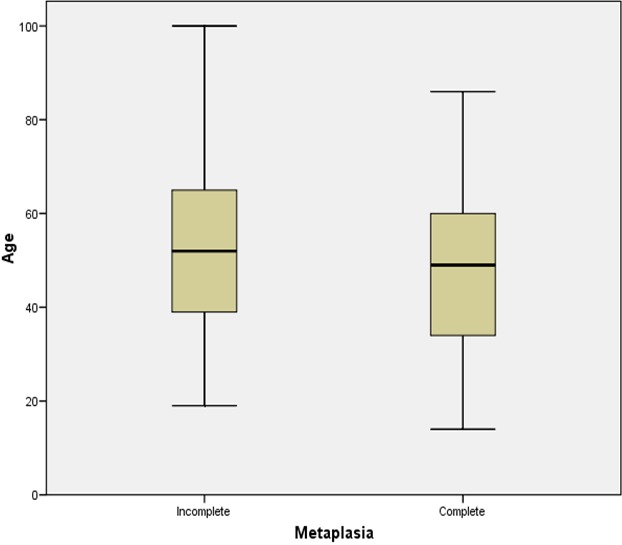
Box plot of patients’ age with complete and incomplete intestinal metaplasia

Within the period under review a total of 775 cases of chronic gastritis were histologically diagnosed. However, only 165 cases (21.3%) showed histological features of intestinal metaplasia of which 137 were properly classified into complete or incomplete intestinal metaplasia. The other 28 biopsies were difficult to characterize due to severe crushing and fragmentation of the tissue sections. The mean age for the occurrence of intestinal metaplasia in gastric biopsies with background chronic gastritis was 50.3 years ± 17.1 SD while the ages of the patients ranged from 10 -100 years (Figure 1). Table 1 shows the age distribution of the subtypes of intestinal metaplasia. Although the specific types of intestinal metaplasia were not significantly associated with the age of the patients (P=0.934), most of the cases of intestinal metaplasia occurred between the ages of 30-59 years. There were 83 males (50.3%) with a mean age of 48.1 years ± 18.2 SD and a 95% Confidence Interval (CI) of 44.1-52.1 years. There were, however, 82 females (49.7%) with a mean age of 52.5 years ± 15.8 SD and a 95% CI of 49.0-56.0 years. Thus the male to female ratio was approximately 1:1. Figure 2 is a box and whisker chart showing the difference in these means and the variation around the mean. There was however, no statistically significant difference in the mean ages of both sexes (t-test of equality of means, P=0.10). There were 78 (47.3%) and 59 (35.8%) cases of incomplete and complete metaplasia respectively. The mean age of all the cases of incomplete intestinal metaplasia was 52.5 ± 16.6 years while that of complete intestinal metaplasia was 48.9 ± 17.5 years (Figure 3). There was no statistically significant difference in the mean ages for the sub-types of intestinal metaplasia (t-test of equality of means, P=0.217). Males accounted for 37 cases (47.4%) of incomplete metaplasia, while females accounted for 41 cases (52.6%). On the other hand, males accounted for 33 cases (55.9%) of complete metaplasia while females accounted for 26 cases (44.1%). histological features of intestinal metaplasia of which 137 were properly classified into complete or incomplete intestinal metaplasia. There was, however, no statistically significant association between the patients’ sex and the sub-type of intestinal metaplasia (P=0.325). Mild, moderate and marked chronic gastritis were respectively found in 10 (13.2%), 25 (32.9%) and 41 (53.9%) cases of incomplete intestinal metaplasia on one hand and in 3 (5.2%), 23 (39.7%) and 32 (55.2%) cases of complete intestinal metaplasia on the other hand. The severity of the inflammation could not be determined in 3 of the cases with intestinal metaplasia due to inadequate histologic material. The type of intestinal metaplasia was not significantly associated with the severity of chronic inflammation of the gastric mucosa (P=0.274). Mild, moderate and marked gastric glandular atrophy were found respectively in 28 (13.0%), 31 (40.3%) and 8 (10.4%) cases of incomplete intestinal metaplasia and in 13 (23.2%), 24 (42.9%) and 10 (17.9%) cases of complete intestinal metaplasia. The type of intestinal metaplasia was however, not significantly associated with the degree of gastric glandular atrophy (P=0.331). There were only 5 cases (3.0%) with moderate dysplasia.

**Table 1:** Age distribution of the subtypes of metaplasia

Age group	Metaplasia		Total
	Incomplete	Complete	
10-19	1(1.3%)	1(1.7%)	2(1.5%)
20-29	7(9.0%)	6(10.2%)	13(9.5%)
30-39	12(15.4%)	13(22.0%)	25(18.2%)
40-49	14(17.9%)	10(16.9%)	24(17.5%)
50-59	15(19.2%)	13(22.0%)	28(20.4%)
60-69	15(19.2%)	7(11.9%)	22(16.1%)
70-79	9(11.5%)	6(10.2%)	15(10.9%)
80-89	4(5.1%)	3(5.1%)	7(5.1%)
>90	1(1.3%)	0(0%)	1(0.7%)
Total	78 (100%)	59(100%)	137(100%)
(Chi-Square P=0.934)			

## Discussion

From this study it is evident that the there is no statistically significant difference in the clinical and histo-morphologic characteristics of complete and incomplete intestinal metaplasia. This study showed that there was no statistically significant association between the distinct subtypes of intestinal metaplasia and the patients’ age, sex, and the various histopathological parameters that characterize chronic gastritis as seen in their gastric biopsies. This study showed that the mean ages for patients with complete and incomplete intestinal metaplasia were similar. This probably suggests that both subtypes of intestinal metaplasia develop within similar time frames from the onset of chronic atrophic gastritis in these patients. Moreover, the fact that the degree of gastric glandular atrophy as well as the severity of the chronic mucosal inflammation did not significantly differ in patients with complete and incomplete intestinal metaplasia alsos uggest that similar pathogenetic mechanisms may underlie these changes. Although various opinions exist about the natural history for the development and progression of complete and incomplete intestinal metaplasia, our study, like many others, seems to suggest that it is highly probable that both subtypes may be quite similar in this regard histological features of intestinal metaplasia of which 137 were properly classified into complete or incomplete intestinal metaplasia [5, 6, 9, 10]. Our finding also supports the view that at the present moment it may not be clinically relevant to differentiate between complete and incomplete intestinal metaplasia [10]. Nevertheless, further studies need to be done in this geographic region to evaluate the relative risk for each subtype of intestinal metaplasia for the development of gastric carcinoma before final conclusions can be arrived at.

Sabageh *et al* working at the same centre where this study was carried out had earlier reported the average age of patients with the intestinal type of gastric carcinomas to be 55 years [11]. This mean age is about 7 years older that the mean ages of patients with both complete and incomplete intestinal metaplasia as shown in this study. This may probably reflect the average time frame it takes for patients with chronic atrophic gastritis and intestinal metaplasia to progress to gastric carcinoma. This, therefore, suggests the need for a more vigorous and early screening program for patients with chronic atrophic gastritis and especially those who develop intestinal metaplasia in the course of the disease so as to forestall progression to gastric carcinoma. The mean age of males with intestinal metaplasia did not differ significantly from that of the female patients. This probably suggests similar aetiopathogenic mechanisms for chronic gastritis and therefore intestinal metaplasia in both sexes. It is possible that hormonal influences (including pregnancy) probably have little or no effect on the rate of development and progression of chronic gastritis and intestinal metaplasia [17]. Earlier reports from our centre have Earlier reports from our centre have shown that the male to female ratio of the intestinal type of gastric carcinoma was 1.2:1 in contrast to the diffuse type of gastric carcinoma which showed an equal sex distribution [11, 12]. In fact, Sabageh et al in an earlier study found this slightly increased frequency of gastric carcinoma in males compared to females [11]. Our study, however, showed an equal sex distribution for intestinal metaplasia. This may suggest that there is a slightly increased likelihood of intestinal metaplasia progressing to gastric carcinoma in male patients. This perhaps may be due to certain, yet unknown, factors restricted to the male sex. It is necessary to conduct a more detailed study to elucidate the statistical significance of the differences between both sexes and the possible role that gender plays (if any) in the transition from intestinal metaplasia to gastric carcinoma. This study also showed that majority (56.1%) of chronic gastritis patients with intestinal metaplasia were between the ages of 30 and 60 years. The mean age from this study is much lower than those from other parts of the world. [7] It is possible that majority of our patients develop chronic gastritis at much younger ages. It is, therefore, necessary that screening, monitoring and appropriate treatments and follow- up programs are commenced as early as possible in these patients even in those younger than 30 years old. Interestingly, Sabageh *et a*l in an earlier study from our centre reported that about 14% of the patients with the intestinal type of gastric carcinoma were younger than 40 years old [11]. Our study, however, found that 29.2% of patients with intestinal metaplasia were younger than 40 years old while 11.0% were below the age of 30 years. The existence of intestinal metaplasia in these very young groups of patients shows that it probably plays an important role in the pathogenesis of intestinal type of gastric carcinoma in such patients who develop gastric carcinoma before the age of 40 years. This observation further buttresses our earlier suggestion of an average transition period of 7 years between intestinal metaplasia and the development of gastric carcinoma. A large cohort study of patients with varying degrees of chronic gastritis may be required to further characterize this transition to gastric carcinoma. Although many studies have shown that the prevalence of intestinal metaplasia increases with age, our study showed that the frequency of cases of intestinal metaplasia decreased with advancing age [17]. This lower frequency among the older subjects may not be due to a reduction in the incidence of intestinal metaplasia, but may actually reflect the Nigerian population statistics which according to the 2006 population census show a significantly lower proportion of individuals in these older age groups [18].

## Conclusion

There are no statistically significant differences between the clinical and histo-morphologic characteristics of complete and incomplete intestinal metaplasia in patients with chronic atrophic gastritis. It probably takes about 7 years for gastric carcinoma to develop in patients with intestinal metaplasia occurring in a background of chronic atrophic gastritis. Since a significant number of our patients develop intestinal metaplasia at an early age, early screening, monitoring and treatment programs are advocated.

### What is known about this topic

- Intestinal metaplasia, a well recognized sequel to chronic atrophic gastritis, increases the risk for the development of gastric adenocarcinoma.
- There are divergent views wirh regards to the relative risks of complete and incomplete intestinal metaplasia for the development of gastric adenocarcinoma.
- There is paucity of information on the clinicopathologic characteristics of intestinal metaplasia among Nigerian patients.

### What this study adds

- Intestinal metaplasia develops at a much earlier age in Nigerian patients.
- Progression from intestinal metaplasia to the development of gastric adenocarcinoma probably takes an average of 7 years in Nigerian patients.
- There are no statistically significant differences in the clinicopathologic characteristics of complete and incomplete intestinal metaplasia.
